# *Candida* spp. colonization: a genotype source found in blood cultures that can become widespread

**DOI:** 10.3389/fcimb.2024.1468692

**Published:** 2024-11-07

**Authors:** Aina Mesquida, Pablo Martín-Rabadán, Luis Alcalá, Almudena Burillo, Elena Reigadas, Patricia Muñoz, Jesús Guinea, Pilar Escribano

**Affiliations:** ^1^ Clinical Microbiology and Infectious Diseases, Hospital General Universitario Gregorio Marañón, Universidad Complutense de Madrid, Madrid, Spain; ^2^ Instituto de Investigación Sanitaria Gregorio Marañón, Madrid, Spain; ^3^ Centro de Investigación Biomédica en Red (CIBER) Enfermedades Respiratorias-CIBERES (CB06/06/0058), Madrid, Spain; ^4^ Medicine Department, Faculty of Medicine, Universidad Complutense de Madrid, Madrid, Spain; ^5^ Facultad HM de Ciencias de la Salud, Universidad Camilo José Cela, Madrid, Spain

**Keywords:** *Candida*, candidemia, colonization, microsatellite, genotyping

## Abstract

**Objective:**

Our previous genotyping studies suggest that some anatomical locations act as reservoirs of genotypes that may cause further candidemia, since we found identical genotypes in gastrointestinal tract or catheter tip isolates and blood cultures, in contrast, we did not find blood culture genotypes in vagina samples. We observed that some genotypes can be found in blood cultures more frequently than others, some of them being called widespread genotypes because have been found in unrelated patients admitted to different hospitals. The presence of widespread genotypes may be more frequently found because of their predisposition to cause candidemia. It is unclear whether genotypes colonizing other anatomical sites different from the gastrointestinal tract can also be detected in this way; we studied *C. albicans*, *C. parapsilosis*, and *C. tropicalis* colonizing genotypes to assess what proportion could be found in blood cultures and the proportion of widespread genotypes.

**Methods:**

The isolates (n= 640 *Candida* isolates from 323 patients) studied herein were obtained from samples processed at the Clinical Microbiology and Infectious Diseases Department of the Gregorio Marañón Hospital (Madrid, Spain) from July 1, 2016, to June 30, 2019. *C. albicans* (n=486), *C. parapsilosis* (n=94), and *C. tropicalis* (n=60) isolates were genotyped using species-specific microsatellite markers and sourced from blood (n=120) and colonized anatomical sites (n=520; catheter [n=50], lower respiratory tract [n=227], skin/mucosa [n=132], and urinary tract [n=111]). Isolates with identical genotypes were those presenting the same alleles for all markers or with only differences at one locus of a given marker. Identical genotypes were further classified as a match (identical genotype found in different groups of samples from a given patient) or as a cluster (identical genotype found in ≥2 patients). Finally, singletons were genotypes detected once. The genotypes found were then compared with our in-house database containing 587 blood genotypes from patients admitted to the Gregorio Marañón Hospital (2007-2023) to assess the proportion of genotypes found in colonized samples that were also found in blood cultures. Moreover, since some of our in-house database genotypes had been tagged as widespread genotypes, we compared the proportions of widespread genotypes as well as the proportions of matches, clusters, and patients involved in clusters found among exclusively colonizing genotypes, exclusively blood culture genotypes, and both colonizing and blood culture genotypes using a standard binomial method.

**Results:**

Intra-patient analysis was conducted exclusively on those patients (n=225; 69.7%) who had ≥2 isolates from a given species; the proportion of patients with matches was lower in exclusively colonized patients than in patients with candidemia and colonizing genotypes (87.3% vs. 94.1%; *p* = 0.126). Inter-patient analysis was conducted considering all patients (n=323) and isolates from groups 1, 2, and 3 (n=640). Overall, we detected 341 genotypes, of which 320 were singletons and 21 were clusters (6.16%). Clusters involving blood cultures and colonizing isolates sourced from catheter tips (14.6%), skin and mucosa (7.5%), urine (7.4%), and lower respiratory tract (4.6%). Cluster-involved patients had not been admitted to the same ward at the same time. Of the 290 colonizing genotypes, 91 (31.1%) were also found in blood cultures, the highest proportion being *C. parapsilosis* (*p* < 0.05); proportions of identical genotypes found in blood cultures and catheter tips were higher than those found in blood cultures and other colonized samples (79.2% vs. 26.7%; *p* < 0.001). Widespread genotype ratios were significantly higher among genotypes found in both blood and colonized samples than among genotypes found exclusively in either blood culture or other colonizing genotypes (31.9% vs. 7.1% vs. 3.7%, respectively; *p* < 0.001).

**Conclusion:**

We observed that 94% of patients with candidemia were colonized by a genotype causing the infection; likewise, a total of 31% of colonizing genotypes were detectable in blood cultures. Finally, identical genotypes found in both colonized samples and blood cultures had a higher probability of being widespread.

Part of this study was partially presented at the 29^th^ European Congress of Clinical Microbiology and Infectious Diseases (ECCMID; P2178 and P2179), Amsterdam, Netherlands 2019.

## 1 Introduction

Infections caused by *Candida* can be either superficial (skin, nails, oropharynx, and vagina) or systemic (invasive candidiasis that involves the bloodstream with or without deep involvement) (Ostrosky-Zeichner and Pappas, 2006). Candidemia is the most common manifestation of invasive candidiasis and is caused by superficial colonizing isolates, including catheter tip or gastrointestinal tract isolates (Nucci and Anaissie, 2001; Leon et al., 2009; Miranda et al., 2009; Lau et al., 2015; Hallen-Adams and Suhr, 2017).

Previously, we found identical genotypes in gastrointestinal tract or catheter tip isolates and blood cultures (Escribano et al., 2014; Mesquida et al., 2023). In contrast, we did not find blood culture genotypes in vagina samples (Mesquida et al., 2021). Our previous genotyping studies suggest that some anatomical locations act as reservoirs of genotypes that may cause further candidemia (Escribano et al., 2014; Mesquida et al., 2021; Mesquida et al., 2023).

The clinical significance of *Candida* isolation from superficial samples (including mucosa), lower and upper respiratory tract samples, and urine samples is commonly regarded as controversial (Fanello et al., 2006; Eggimann et al., 2015; Jensen et al., 2015; Pendleton et al., 2017). *Candida* colonizing isolates cause invasive infections when the patient’s risk factors predispose them to candidemia (Leon et al., 2009; Leon et al., 2014); alternatively, some colonizing genotypes may have the ability to cause candidemia. However, genotyping studies focusing on patients with candidemia who had previous colonization are not only scarce but also limited by the low number of isolates studied and/or by the use of poorly discriminatory molecular techniques (Nucci and Anaissie, 2001; Chaves et al., 2012; Jensen et al., 2015; Li et al., 2016).

We previously observed that some genotypes can be found in blood cultures more frequently than others, some of them being called widespread genotypes because have been found in unrelated patients (Guinea et al., 2020; Diaz-Garcia et al., 2022b; Mesquida et al., 2023). The presence of widespread genotypes is unclear; however, it could be hypothesized that they are more frequently found because of their predisposition to cause candidemia. In line with this, we previously reported that the higher the probability of a rectal genotype being found in blood cultures, the higher the odds of it being a widespread genotype (Mesquida et al., 2023). However, the proportion of widespread genotypes in non-rectal colonizing isolates is unknown.

We studied *C. albicans*, *C. parapsilosis*, and *C. tropicalis* colonizing genotypes to assess what proportion could be found in blood cultures and the proportion of widespread genotypes.

## 2 Materials and methods

### 2.1 Isolates collected and studied

**Figure 1:**
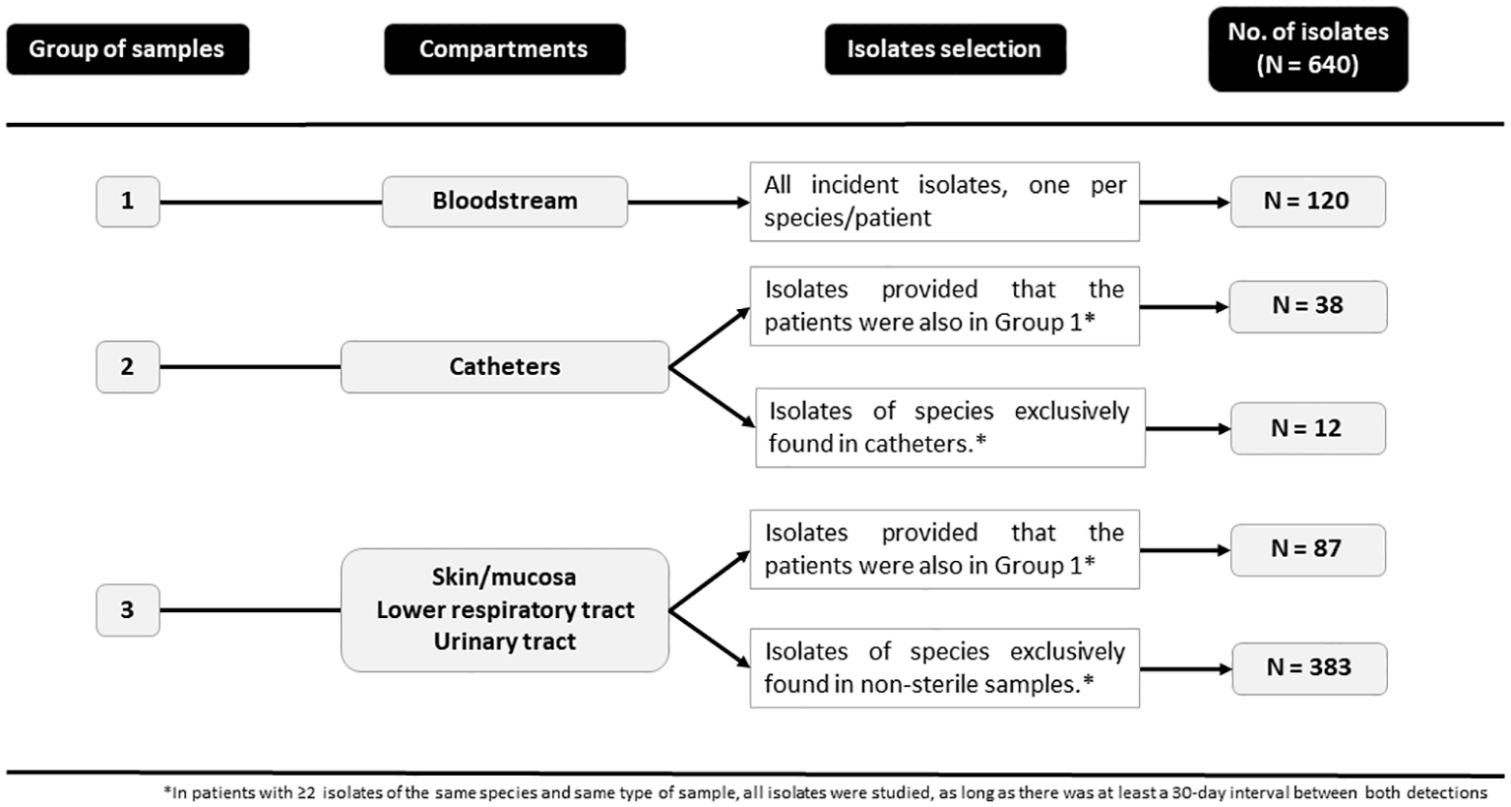
Isolates and selection criteria from each study group.

From July 1, 2016, to June 30, 2019 all consecutive (and available) isolates (n=640) collected at the Clinical Microbiology and Infectious Diseases Department of the Gregorio Marañón Hospital (Madrid, Spain) were here studied. Briefly, isolates were sourced from three groups of samples: group 1 (blood cultures), group 2 (catheter tips), and group 3 (skin/mucosa [excluding vaginal exudates], lower respiratory tract, and urinary tract). A total of 323 patients had either isolates from group 1 and, therefore, had candidemia (n=120; 68 of whom also had isolates from groups 2 and/or 3) or from groups 2 and/or 3 (n=203; 157 of whom had ≥2 isolates from the same species). Isolates from groups 2 and/or 3 were called colonizing isolates/genotypes; we did not conduct prospective screening of *Candida* colonization in critically ill patients. Isolate distributions among species and sample groups and details of the source of the isolates and selection criteria are described in **Table 1** and **Figure 1**.

**Table 1:** Number of patients (isolates) studied and distributed by clinical source (groups 1, 2, 3, or combinations of them).

Species	Patients with candidemia				Patients without candidemia			Overall
	G1	G1+G2	G1+G3	G1+G2+G3	G2	G2+G3	G3	
*C. albicans*	23 (23)	10 (20)	22 (67)	13 (50)		6 (16)	164 (310)	**238 (486)**
*C. parapsilosis*	25 (25)	9 (18)	3 (6)	4 (14)	1 (1)	3 (11)	11 (19)	**56 (94)**
*C. tropicalis*	4 (4)	1 (2)	5 (11)	1 (4)		1 (3)	17 (36)	**29 (60)**
**Overall**	**52 (52)**	**20 (40)**	**30 (84)**	**18 (68)**	**1 (1)**	**10 (30)**	**192 (365)**	**323 (640)**

G1: group 1; G2: group 2; G3: group 3; Isolates from G3 were sourced from the lower respiratory tract (n=227), skin and mucosa (n=132), or urine (n=111). Bold numbers indicate the overall data.

### 2.2 Genotyping data analysis

Isolates were genotyped using species-specific microsatellite markers to detect *C. albicans* (CDC3, EF3, HIS3 CAI, CAIII, and CAVI), *C. parapsilosis* (CP1, CP4a, CP6, and B), and *C. tropicalis* (Ctrm1, Ctrm10, Ctrm12, Ctrm21, Ctrm24, and Ctrm28) (Botterel et al., 2001; Sampaio et al., 2005; Sabino et al., 2010; Vaz et al., 2011; Wu et al., 2014). PCR products underwent electrophoresis using an ABI 3730XL Analyzer and electropherograms were analyzed with GeneMapper v.4.0 software (Applied Biosystems-Life Technologies Corporation, California). Genetic relationships between genotypes were studied by constructing a minimum spanning tree using BioNumerics version 7.6 (Applied Maths, Sint-Martens-Latem, Belgium). Isolates with identical genotypes were those presenting the same alleles for all markers or with only differences at one locus of a given marker (Mesquida et al., 2024). Identical genotypes were further classified as a match (identical genotype found in different groups of samples from a given patient) or as a cluster (identical genotype found in ≥2 patients). Finally, singletons were genotypes detected once.

The genotypes found were then compared with our in-house database containing 587 blood genotypes from patients admitted to the Gregorio Marañón Hospital (2007–2023) to assess the proportion of genotypes found in colonized samples that were also found in blood cultures. Moreover, since some of our in-house database genotypes had been tagged as widespread genotypes (clusters involving isolates from patients admitted to different hospitals) (Guinea et al., 2020; Diaz-Garcia et al., 2022b), we compared the proportions of widespread genotypes found among exclusively colonizing genotypes, exclusively blood culture genotypes, and both colonizing and blood culture genotypes.

The proportion of genotypes, matches, clusters, patients involved in clusters, and widespread genotypes were compared using a standard binomial method (95% confidence intervals; Epidat 3.1 software, Servicio de Información sobre Saúde Pública de la Dirección Xeral de Saúde Pública de la Consellería de Sanidade, Xunta de Galicia, Spain).

### 2.3 Ethical considerations

This study was approved by the Ethics Committee of the Gregorio Marañón Hospital (CEim; study no. MICRO.HGUGM.2020-012).

## 3 Results

### 3.1 Intra-patient genotype analysis (matches)

Intra-patient analysis was conducted exclusively on those patients (n=225; 69.7%) who had ≥2 isolates from a given species. A total of 157 patients only had colonizing isolates (mean: 2.23; range: 2 to 6 per patient) sourced from the respiratory tract, urine, skin and mucosa, and catheter tips. We observed matches in 87.3% (n=137/157) of the patients, whereas the remaining patients (n=20) had non-matching genotypes involving mostly lower respiratory tract isolates (n=19) (**Table 2**).

**Table 2:** Intra-patient analysis of genotypes; only patients with two or more isolates of the same *Candida* spp. are shown.

	*C. albicans*	*C. parapsilosis*	*C. tropicalis*	Overall
No. of patients with candidemia and colonization				
Total of patients	**45**	**16**	**7**	**68**
Patients with matches	43	15	6	64
Patients without matches	2	1	1	4
No. of patients with colonization exclusively				
Total of patient/genotypes	**131**	**9**	**17**	**157**
Patients with matches	116	9	12	137
Patients without matches	15	0	5	20
**Overall**	**176**	**25**	**24**	**225**

Bold numbers indicate the overall data.

Moreover, 68 patients had candidemia plus colonizing isolates (mean: 1.8; range: 1 to 5 per patient). Of these, 29 patients had colonizing isolates only from group 3 samples, 20 patients had isolates from catheter tips, and 19 patients had isolates from catheter tips + other group 3 samples. In 64 of these 68 patients (94.1%), we observed matches between blood culture and colonizing genotypes sourced from catheter tips (n=38/38; 100%), urine (n=23/25; 92%), skin and mucosa (n=15/17; 88.2%), and lower respiratory tract (n=36/44; 81.8%) (**Supplementary Table S1**). In the remaining four patients without matches, the colonizing isolates came from urine, skin and mucosa, or the lower respiratory tract, and were collected before (one patient infected by *C. tropicalis*) or after (two patients infected by *C. albicans* and one by *C. parapsilosis*) the candidemia diagnosis (**Supplementary Table S1**).

As for *C. albicans*, 43/45 patients had blood culture genotypes matching colonizing genotypes isolated before the candidemia diagnosis (range: 2-29 days; six patients), after the candidemia diagnosis (range: 0-231 days; 27 patients), or both before and after blood culture isolation (range: -119 days to +90 days from the candidemia diagnosis; 10 patients). As for *C. parapsilosis*, 15/16 patients had blood culture genotypes matching colonizing genotypes isolated before the candidemia diagnosis (71 days; one patient), after the candidemia diagnosis (range: 1-92 days; 12 patients), or both before and after blood culture isolation (range: -14 days to +2 days from the candidemia diagnosis; two patients). As for *C. tropicalis*, 6/7 patients had blood culture genotypes matching colonizing genotypes isolated before the candidemia diagnosis (range: 2-32 days; three patients), after the candidemia diagnosis (one day; one patient) or both before and after blood culture isolation (range: -28 days to +7 days from the candidemia diagnosis; two patients). In 24/68 patients (35.3%), the colonizing genotypes matching blood culture genotypes were detected a mean of 21 days before candidemia onset, and in 20 cases, the genotype was found in the lower respiratory tract.

The proportion of patients exclusively colonized and presenting genotypes matching different colonized sample groups tended to be lower than the proportion of patients presenting genotypes matching colonized samples and blood cultures (87.3% vs. 94.1%; *p* = 0.126).

### 3.2 Inter-patient genotype analysis (clusters)

Inter-patient analysis was conducted considering all patients (n=323) and isolates from groups 1, 2, and 3 (n=640). The genotype distributions among species and isolate groups are described in **Table 3**. Overall, we detected 341 genotypes, of which 320 were singletons and 21 were clusters (6.16%) involving isolates from group 1 exclusively (n=1/341; 0.3%), from group 3 exclusively (n=7/341; 2%), from groups 1 + 2 and/or 3 (n=12/341; 3.5%), and groups 2 and 3 (n=1/341; 0.3%). Clusters involving blood cultures and colonizing isolates sourced from catheter tips (n=7/48 genotypes; 14.6%), skin and mucosa (n=8/106 genotypes; 7.5%), urine (n=7/94 genotypes; 7.4%), and lower respiratory tract (n=8/173 genotypes; 4.6%). Cluster-involved patients had not been admitted to the same ward at the same time.

**Table 3:** Inter-patient analysis of genotypes, distribution among species, and group of samples.

Species (and kind of genotypes)	No. of genotypes per group of samples							Overall
	G1	G2	G3	G1+G2	G1+G3	G1+G2+G3	G2+G3	
*C. albicans*								
No. of genotypes	24	2	180	12	18	10	5	**251**
No. of singleton	23	2	174	12	15	6	5	**237**
No. of clusters (No. of patients involved; range of patients per cluster)	1 (2; 2)	0	6 (18; 2-6)	0	3 (15; 3-9)	4 (14; 2-5)	0	**14 (49; 2-9)**
*C. parapsilosis*								
No. of genotypes	21	2	13	7	4	5	2	**54**
No. of singleton	21	2	13	7	2	2	2	**49**
No. of clusters (No. of patients involved; range of patients per cluster)	0	0	0	0	2 (8; 2-6)	3 (8; 2-4)	0	**5 (16; 2-6)**
*C. tropicalis*								
No. of genotypes	6	0	24	1	3	1	1	**36**
No. of singletons	6	0	23	1	3	1	0	**34**
No. of clusters (No. of patients involved; range of patients per cluster)	0	0	1 (2; 2)	0	0	0	1 (2; 2)	**2 (4; 2)**
**Overall genotypes**	**51**	**4**	**217**	**20**	**25**	**16**	**8**	**341**

G1: group 1; G2: group 2; G3: group 3; Genotypes from G3 were sourced from the lower respiratory tract (n=173), skin and mucosa (n=106), and urine (n=94). Bold numbers indicate the overall data.

A total of 14 (5.6%) *C. albicans* genotypes were clusters and involved 20.6% of patients harboring *C. albicans* (range: 2 to 9 patients/cluster); clusters involved isolates from group 1 exclusively (n=1/14; 7.1%), from group 3 exclusively (n=6/14; 42.9%), or group 1 + groups 2 and/or 3 (n=7/14; 50%). A total of five *C. parapsilosis* genotypes were clusters and involved 28.6% of patients harboring *C. parapsilosis* (range: 2 to 6 patients/cluster); clusters involved isolates from group 1 + groups 2 and/or 3 (n=5/5; 100%). Finally, a total of two *C. tropicalis* genotypes were clusters and involved 13.8% of patients harboring *C. tropicalis*; clusters involved two patients each and involved isolates from groups 2 and/or 3 (n=2/2; 100%) (**Table 3**). None of these differences reached statistical significance for the proportion of clusters and patients involved in a cluster by species (*p* > 0.05).

### 3.3 Comparisons between colonizing genotypes and blood culture genotypes. Proportions of widespread genotypes

The colonizing genotypes found herein were then compared with blood culture genotypes, not only from group 1 but also those retrieved from our database. Of the colonizing genotypes found, 31.1% (91/290) were identical to those found in blood cultures; *C. parapsilosis* being the species with the highest proportion (57.6%; *p* < 0.01; **Table 4**). The proportion of identical genotypes found in blood cultures and catheter tips was higher than that found in blood cultures and group 3 isolates (38/48 genotypes [79.2%] vs. 71/266 genotypes [26.7%]; *p* < 0.001). The group 3 genotypes also found in blood cultures were sourced from the following locations (and accounted for the corresponding proportion within the genotypes from these locations): the lower respiratory tract (n=50/173; 28.9%), skin and mucosa (n=33/106; 31.1%), and urine (n=34/94; 36.2%; *p* > 0.5). Similar observations were found in the analysis disaggregated per species (**Table 4**).

**Table 4:** Genotypes from colonizing samples also found in blood cultures including all the blood cultures analyzed (587 added).

Species	No. of colonizing genotypes	No. of colonizing genotypes found in blood			
		G1+G2	G1+G3	G1+G2+G3	Overall
*C. albicans*	277	12 (4.3%)	43 (15.5%)	11 (3.9%)	**66 (23.8%)**
*C. parapsilosis*	33	7 (21.2%)	6 (18.2%)	6 (18.2%)	**19 (57.6%)**
*C. tropicalis*	30	1 (3.3%)	4 (13.3%)	1 (3.3%)	**6 (16.6%)**
**Overall**	**290**	**20 (6.9%)**	**53 (18.3%)**	**18 (6.2%)**	**91 (31.4%)**

G1: group 1; G2: group 2; G3: group 3. Bold numbers indicate the overall data.

**Figure 2:**
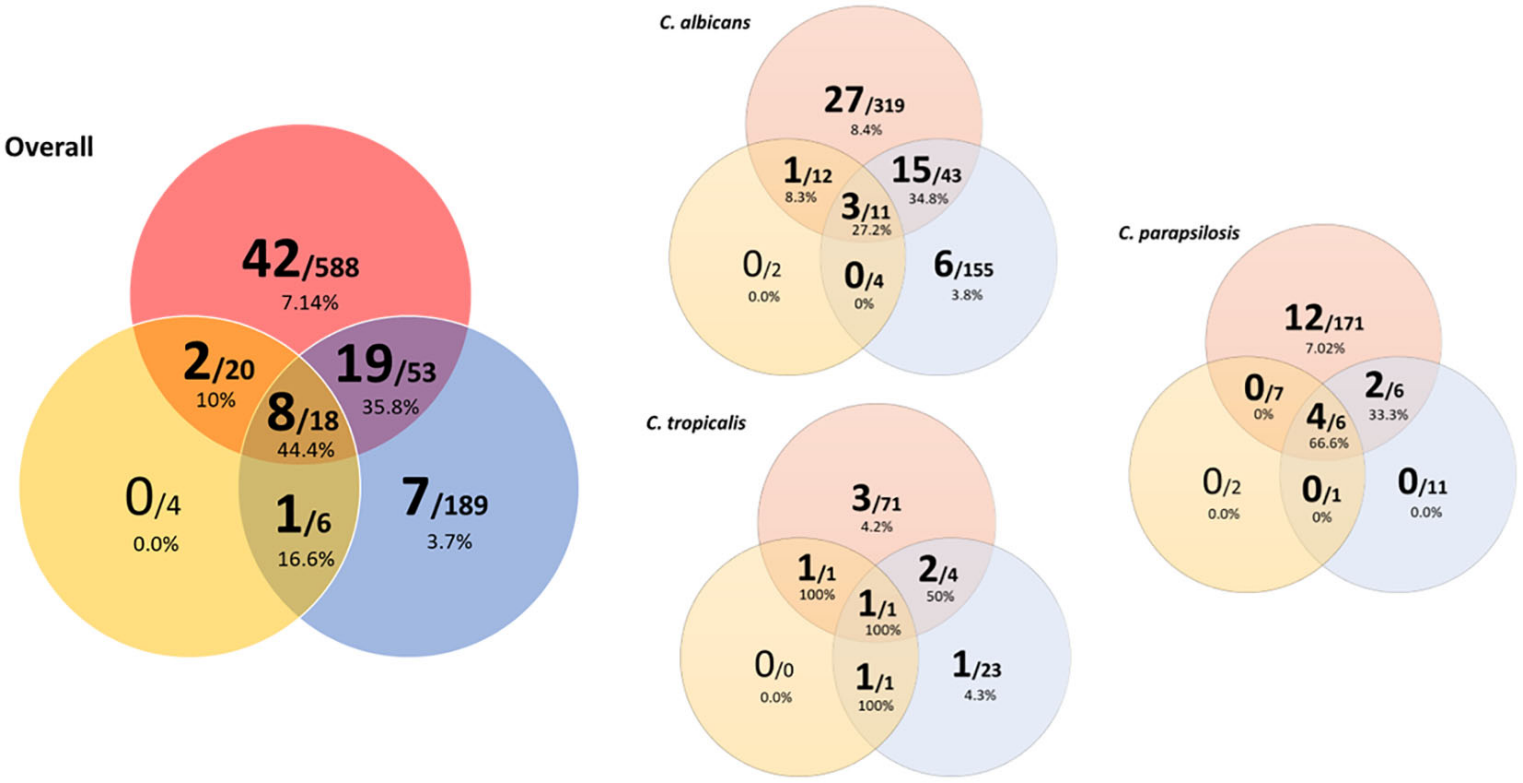
Venn diagram showing widespread clusters involving colonizing samples, and/or blood culture, and/or catheter tip isolates, overall and disaggregated by species. Circle colors indicate the isolate’s clinical source (blood culture: red; catheter tip: yellow; colonizing sample: blue). Numbers in the larger font indicate widespread clusters; numbers in the smaller font indicate total clusters. Percentages indicate the proportions of widespread clusters.

The proportions of widespread genotypes were significantly higher among genotypes found in both blood and colonized samples than among those found exclusively in either blood culture or group 3 isolates (29/91 genotypes [31.9%] vs. 42/588 genotypes [7.1%] vs. 7/189 genotypes [3.7%], respectively; *p* < 0.001). We did not find widespread genotypes exclusively in catheter tips (**Figure 2**). Identical observations were found in the analysis disaggregated per species (**Figure 2**).

## 4 Discussion

In this study, we observed that 94% of patients with candidemia and colonizing isolates were colonized by the genotype causing the infection. Furthermore, 31% of the colonizing genotypes studied were detectable in blood cultures, with catheter tips showing the highest percentage of identical genotypes found in blood cultures. Finally, identical genotypes found in both colonized samples and blood cultures were more likely to be widespread.

Candidemia can have an endogenous source, where the intra-abdominal cavity is a relevant reservoir of *Candida* spp. isolates causing further candidemia. Genotyping may be helpful to illustrate such an infection route. In a previous study, we observed that 15.5% of genotypes found in the rectum were also found in blood cultures and/or intra-abdominal samples (Mesquida et al., 2023). Alternatively, the exogenous sources are colonizing genotypes able to reach the bloodstream. Since patients may become colonized by *Candida* spp., further invasive infections are a consequence of some of the colonizing genotypes reaching the bloodstream. Some score-based procedures have proven useful in the detection of patients at high risk of acquiring candidemia: multifocal *Candida* colonization is one of the predisposing factors to candidemia (Ostrosky-Zeichner and Pappas, 2006; Leon et al., 2009). Our current observations also endorse the exogenous route of infection. Up to 31% of colonizing genotypes (26.7% excluding catheter tip genotypes) were detectable in blood cultures and matches between blood culture and colonizing genotypes were observed in 94% of the patient subset with candidemia plus colonizing isolates. In fact, the colonizing genotypes matching blood genotypes had been detected before candidemia in at least one-third of the patients studied, most of them sourced from the lower respiratory tract. However, we cannot rule out the presence of intra-abdominal genotypes matching those found in blood cultures in the remaining two-thirds of patients given that the retrospective nature of the study prevented us from screening patients for *Candida* colonization. *Candida*-colonized catheter tips are a well-known source of candidemia; we previously found identical genotypes in blood cultures and catheter tips in patients with catheter-related candidemia (Escribano et al., 2013a; Escribano et al., 2014). The high number of matches between blood cultures and catheter tips here reported reinforces this candidemia source. Given that we also observed matches between blood cultures and other types of colonized samples, such as mucosa or the lower respiratory tract, in 40 patients may simply reflect the fact that multicolonization by a given genotype is a risk factor for further development of candidemia caused by the genotype in question. Such observations had been previously reported (Chaves et al., 2012).

The genotyping of blood culture isolates has also been useful in uncovering the presence of *Candida* clusters (identical *Candida* spp. genotypes infecting ≥2 different patients). Clusters may indicate patient-to-patient hospital transmission and thus are useful to trace hospital outbreaks in epidemiologically related patients (Escribano et al., 2018; Diaz-Garcia et al., 2022b). In contrast, some clusters involve non-epidemiologically related patients, and patients may be admitted to different hospitals and cities; we previously called these clusters “widespread” and they do not indicate patient-to-patient transmission (Guinea et al., 2020; Diaz-Garcia et al., 2022b; Diaz-Garcia et al., 2022a). The significance of these widespread clusters is not yet clear, however, they may indicate frequent genotypes: the more frequent a genotype is, the higher the probability of detecting it in blood cultures. We previously found that up to 11% of blood culture genotypes were clusters involving different patients (Escribano et al., 2013b; Guinea et al., 2020); these genotypes may be more suitable as a cause of candidemia than singleton genotypes. In line with this hypothesis, we believe that there might be some genotype reservoirs that could cause candidemia. One of these reservoirs may be the intra-abdominal cavity (endogenous source) that hosts between 6 and 11% of clusters that can cause candidemia (Diaz-Garcia et al., 2022b; Mesquida et al., 2023). Other reservoirs may be mucosa, lower respiratory tract, catheter tips, and urine (exogenous), which host up to 6% of clusters, as shown in the current study. On balance, our data suggest that the aforementioned reservoirs host singleton and cluster genotypes (the latter accounting for up to 6% of genotypes), which can be found to cause candidemia in (unrelated) patients. The fact that the proportion of widespread genotypes tended to be significantly higher when more sample types were involved—as reported here and previously (Diaz-Garcia et al., 2022b; Mesquida et al., 2023)—reinforces the idea that widespread clusters can cause invasive infections.

Our study is subjected to limitations. The main limitation of our study is that the sample collection was retrospective and, therefore, made upon clinical indication, this could introduce bias in sample collection and affect the ability to establish causal relationships between isolates colonization or invasive candidiasis. Furthermore, the study does not fully address potential confounding factors that may influence the observed relationships, such as the underlying health conditions of the patients or prior antibiotic use. Finally, other clinically relevant species such as *C. glabrata* or *C. krusei* were not here studied. Prospective studies involving colonized patients ± invasive infections, and including those ones infected by species other than *C. albicans*, *C. parapsilosis*, and *C. tropicalis*, are needed to address this question.

In conclusion, we observed that 94% of patients with candidemia were colonized by the genotype causing the infection. A total of 31% of colonizing genotypes were detectable in blood cultures, and identical genotypes found in both colonizing samples and blood cultures had a higher probability of being widespread.

## Data Availability

The raw data supporting the conclusions of this article will be made available by the authors, without undue reservation.
